# Supplementary material for: The Class I HDAC Inhibitor Valproic Acid Strongly Potentiates Gemcitabine Efficacy in Pancreatic Cancer by Immune System Activation
Source: Biomedicines. 2022 Feb 22;10(3):517. doi: 10.3390/biomedicines10030517 (PMC8945828; doi:10.3390/biomedicines10030517)
Supplement: Supplementary file 1 [file biomedicines-10-00517-s001.zip › biomedicines-1526453-supplementary.pdf]

biomedicines

Article

The Class I HDAC Inhibitor Valproic Acid Strongly Potentiates Gemcitabine Efficacy in Pancreatic Cancer by Immune System Activation

Amber Blaauboer [1,2], Peter M. van Koetsveld [2], Dana A. M. Mustafa [3], Jasper Dumas [3], Fadime Dogan [2], Suzanne van Zwienen [2], Casper H. J. van Eijck [1] and Leo J. Hofland [2,*]

[1] Department of Surgery, Erasmus Medical Center, 3015 GD Rotterdam, The Netherlands; a.blaauboer@erasmusmc.nl (A.B.); c.vaneijck@erasmusmc.nl (C.H.J.v.E.)
[2] Department of Internal Medicine, Division of Endocrinology, Erasmus Medical Center, 3015 GD Rotterdam, The Netherlands; p.vankoetsveld@erasmusmc.nl (P.M.v.K.); f.dogan@erasmusmc.nl (F.D.); svzwienen@hotmail.com (S.v.Z.)
[3] Department of Pathology, The Tumor Immuno-Pathology Laboratory, Erasmus Medical Center, 3015 GD Rotterdam, The Netherlands; d.mustafa@erasmusmc.nl (D.A.M.M.); j.dumas@erasmusmc.nl (J.D.)
* Correspondence: l.hofland@erasmusmc.nl

Supplementary material

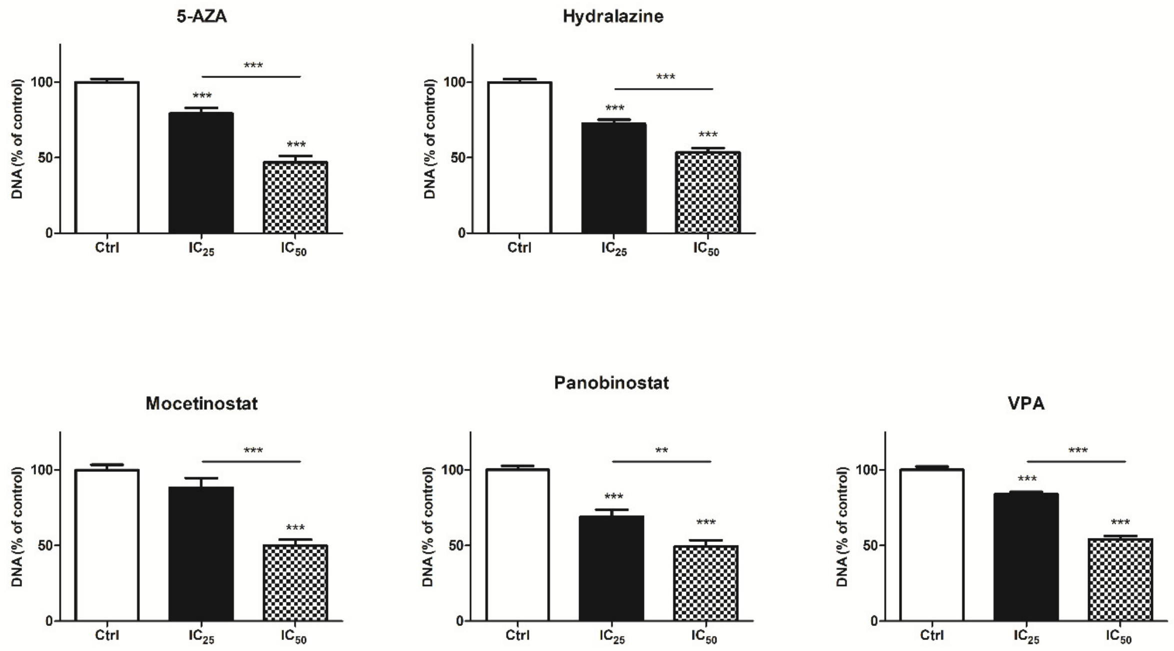

Supplementary Figure S1. Antiproliferative effect of IC_{25} and IC_{50} of the indicated epi-drugs. Values represent mean ± SEM of at least two independent experiments in quadruplicate and are shown as the percentage of control. **p<0.01 and ***p<0.001 versus control.

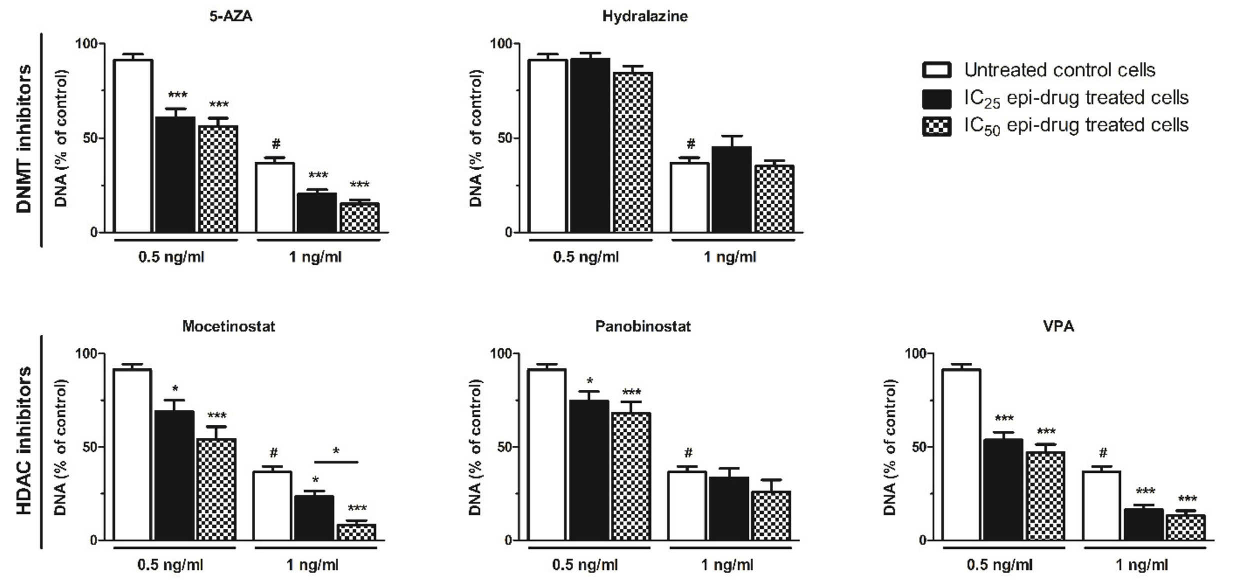

Supplementary Figure S2. Antiproliferative effect of 0.5 ng/ml and 1 ng/ml gemcitabine, with or without epi-drugs. Column bars represent the effect of gemcitabine (0.5 and 1 ng/ml) in untreated control cells (white bar), IC_{25} epi-drug treated cells (black bar), and IC_{50} epi-drug treated cells (black dotted bar) on total DNA, as a measure of cell number, in KPC3 cells after seven days of treatment. Data are presented as percentage of vehicle treated control. For epi-drug treated cells, the effect of epi-drug alone was set on 100% and used as control. Values represent mean ± SEM and are shown as percentage of control. * $p < 0.05$ and *** $p < 0.001$ versus control. $^{\#}$ $p < 0.01$ versus untreated control cells.

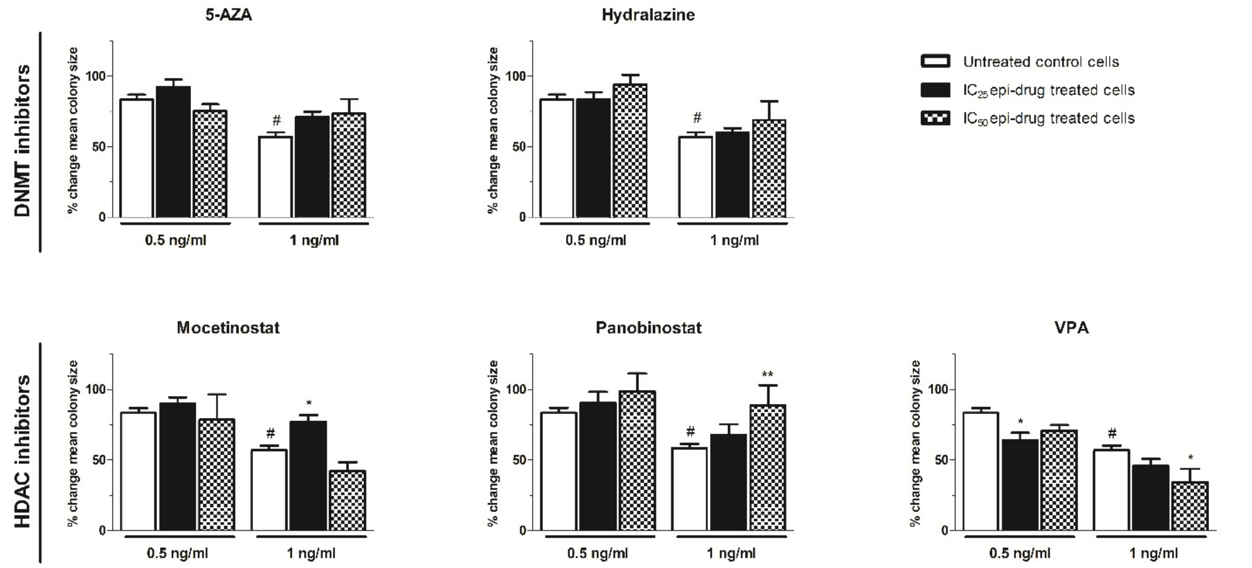

Supplementary Figure S3. Cytostatic effect of gemcitabine, with or without epi-drugs, on colony size. Column bars represent the effect of seven days gemcitabine (0.5 and 1 ng/ml) in untreated control cells (white bar), IC_{25} epi-drug treated cells (black bar), and IC_{50} epi-drug treated cells (black dotted bar) on colony size. Data are presented as percentage of vehicle treated control. For epi-drug treated cells, the effect of epi-drug alone was set on 100% and used as control. Values represent mean ± SEM of at least two independent experiments and are shown as a percentage of control. * p <0.05 and ** p <0.01 versus untreated control cells. $^{\#}$ p <0.05 effect of gemcitabine monotherapy versus control cells.

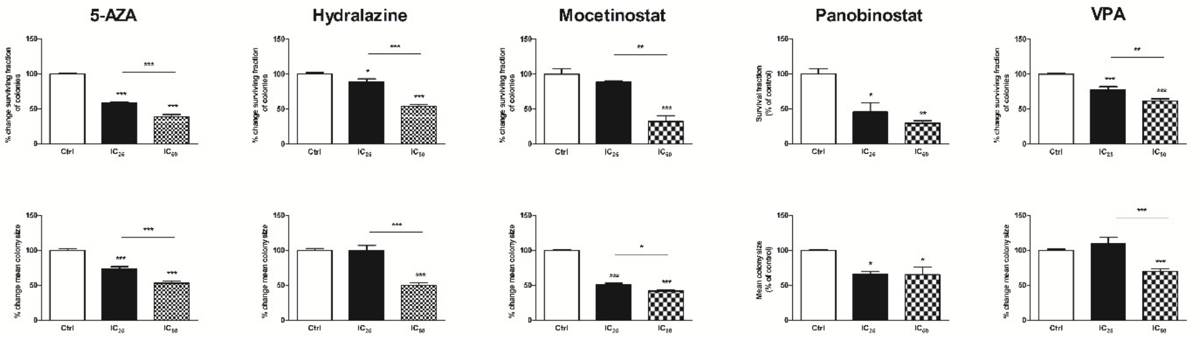

Supplementary Figure S4. Cytotoxic effect (upper panel) and cytostatic effect (lower panel) of the indicated epi-drugs in KPC3 cells. Values represent mean ± SEM of at least two independent experiments in quadruplicate and are shown as the percentage of control. * p <0.05, ** p <0.01, and *** p <0.001 versus control.

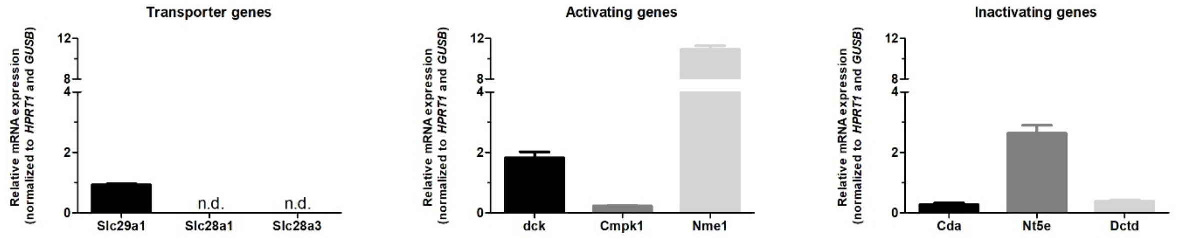

Supplementary Figure S5. Baseline *mRNA* expression of genes involved in gemcitabine transport and metabolism. Values represent relative *mRNA* expression. N.d.; not detectable.

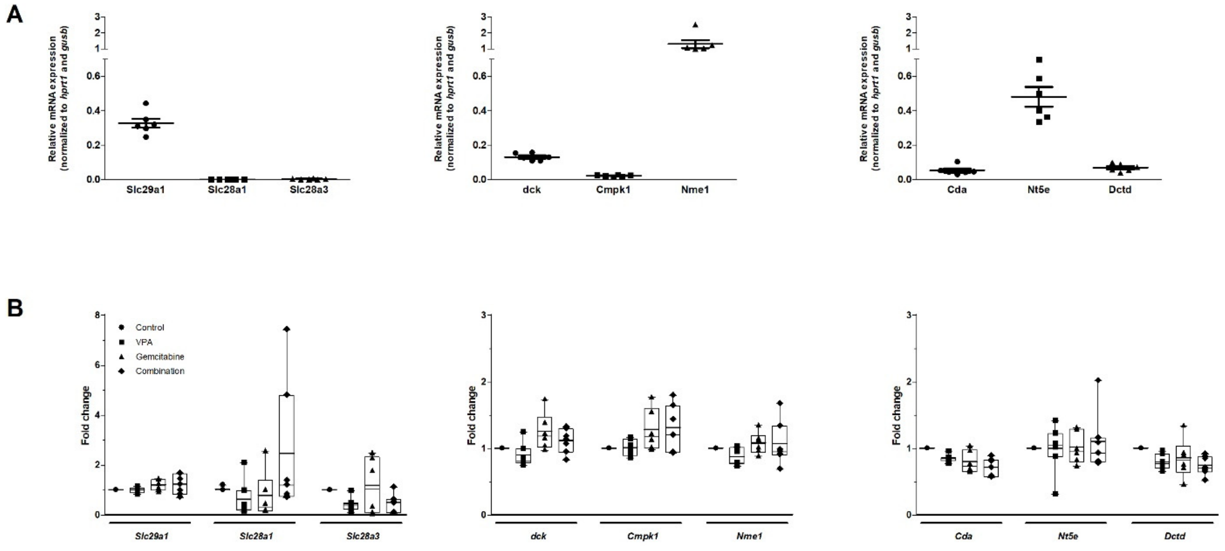

Supplementary Figure S6. Effect of VPA on *mRNA* expression of genes involved in gemcitabine transport and metabolism in KPC3 xenografts. A) Baseline expression and B) Fold change in *mRNA* expression between ● vehicle treated tumors, ■ VPA, ▲ gemcitabine (light grey), and the ◆ combination of VPA plus gemcitabine. Values represent mean ± SEM.

Supplementary Table S1. Primers and probes used for RT-qPCR.

Gene	Assay ID	EF
Hprt1	Mm03024075_m1	1.91
Gusb	Mm01197698_m1	1.98
Slc29a1	Mm01270577_m1	1.98
Slc28a1	Mm01315355_m1	2
Slc28a2	Mm00491586_m1	2
Dck	Mm00432794_m1	2
Cmpk1	Mm01603215_g1	1.8
Nme1	Mm01612215_m1	1.8
Cda	Mm01341706_m1	2
Nt5e	Mm00501910_m1	2
Dctd	Mm00618904_m1	2

All used primers are commercially available (Thermo Fisher Scientific, Breda, the Netherlands). B-actin, Beta-actin; cda, cytidine deaminase; Cmpk1, cytidine monophosphate kinase 1; dck, deoxycytidine kinase; dctd, deoxycytidylate deaminase; EF, efficiency factor; gusb, glucuronidase beta; hprt1, 7 hypoxanthine-guanine phosphoribosyl transferase 1; nme1, nucleoside diphosphate kinase A; nt5e, 5’-nucleotidases; slc28a1, solute carrier family 28 member 1; slc28a3, solute carrier family 28 member 3; slc29a1, solute carrier family 29 member 1.

Supplementary Table S2. List of housekeeping genes used for NanoString analysis.

Gene	Assay ID
Pum1	NM_001159605
Tmub2	NM_028076
Tbp	NM_013684
Polr2a	NM_009089
Sdha	NM_023281
Oaz1	NM_008753
Tbc1d10b	NM_144522
Dnajc14	NM_028873
*Abcf1**	NM_013854
*G6pdx**	NM_008062
*Psmc4**	NM_011874

* Highlighted genes were excluded for NanoString analysis based on expression stability and minimum variance.All used primers are commercially available (NanoString Technology, USA). Pum1, Pumilio RNA Binding Family Member 1; Tmub2, Transmembrane And Ubiquitin Like Domain Containing 2; Tbp, TATA-Box Binding Protein; Polr2a, RNA Polymerase II Subunit A; Sdha, Succinate Dehydrogenase Complex Flavoprotein Subunit A; Oaz1, Ornithine Decarboxylase Antizyme 1; Tbc1d10b, TBC1 Domain Family Member 10B; Dnajc14, DnaJ Heat Shock Protein Family Member C14; Abcf1, ATP Binding Cassette Subfamily F Member 1; G6pdx, glucose-6-phosphate dehydrogenase X-linked; Psmc4, Proteasome 26S Subunit, ATPase 4.

Supplementary Table S3. Significantly differentially expressed genes in gemcitabine (left) and combination (right) treated mice compared to untreated mice.

	Gemcitabine vs control				Combination vs control		
	**Log2 fold change**	**P-value**	**BH.p.value**		**Log2 fold change**	**P-value**	**BH.p.value**
Aldoc	-0.66	0.00244	0.0427	*Aldoc*	-1.06	1.05e-05	0.00244
Bmp2	-1.21	0.000873	0.0315	*Angpt2*	-1.02	0.000925	0.0283
Bnip3	-0.999	0.00224	0.0427	*Axl*	0.676	0.00228	0.0418
Bnip3l	-0.651	0.000196	0.017	*Bnip3l*	-0.773	1.61e-05	0.00244
Cdkn1a	-1.13	5.02e-05	0.0096	*Ccnd2*	-0.897	0.00169	0.0358
Ctsw	1.04	0.001	0.0315	*Cd300a*	0.977	0.00116	0.0298
Dtx4	0.572	0.00242	0.0427	*Clec7a*	1.24	0.000278	0.0121
Dusp1	-0.934	0.000895	0.0315	*Ctsw*	1.31	5.51e-05	0.00436
Eif4ebp1	-0.446	0.000947	0.0315	*Ero1l*	-1.47	6.47e-05	0.00436
Ero1l	-1.49	8.4e-05	0.0096	*Hk1*	-0.438	0.00271	0.0456
Fgf13	2.15	0.00125	0.0315	*Il18bp*	0.88	5.27e-05	0.00436
Fstl3	-0.938	8.24e-05	0.0096	*Il22ra1*	-0.538	0.00179	0.0358
H2-K1	-0.581	0.00244	0.0427	*Irf3*	0.371	0.000593	0.0211
Hey1	-0.865	0.00287	0.0446	*Itgal*	1.04	0.000736	0.0248
Hk1	-0.514	0.000945	0.0315	*Itpk1*	-1.25	7.35e-08	4.46e-05
Hk2	-1.42	0.00108	0.0315	*Lilra5*	2.02	4.73e-05	0.00436
Igf2r	-0.576	0.000761	0.0315	*Mxi1*	-0.658	0.00241	0.043
Irf2	-0.252	0.0024	0.0427	*Ndufa4l2*	-1.28	0.00123	0.0298
Itpk1	-1	3.79e-06	0.0023	*Nfkb2*	0.345	0.00218	0.0413
Jag2	-0.493	0.00284	0.0446	*P4ha1*	-0.64	0.00097	0.0283
Lilra5	1.43	0.00242	0.0427	*P4ha2*	-0.915	0.00012	0.00664
Mdm2	-0.408	0.00314	0.0476	*Pck2*	-0.339	0.0014	0.0315
Mtor	-0.253	0.00269	0.0441	*Pdk1*	-0.95	1.3e-05	0.00244
Mxi1	-0.705	0.00185	0.0427	*Prkca*	-0.68	0.000134	0.00676
Ndufa4l2	-1.24	0.00228	0.0427	*Rad51c*	-0.561	0.00131	0.0305
Nfil3	-0.572	0.00236	0.0427	*Rbl2*	-0.339	0.00183	0.0358
Nos2	-1.85	0.00117	0.0315	*Rpl23*	-0.908	6.44e-05	0.00436
P4ha1	-0.65	0.00118	0.0315	*Rps6kb1*	-0.367	0.00103	0.0283
P4ha2	-1.03	4.9e-05	0.0096	*Sfrp1*	1.78	0.000251	0.0117
Pdk1	-0.766	0.000262	0.0199	*Srebf1*	-0.483	0.000103	0.00622
Pfkfb3	-0.645	0.000762	0.0315	*Stat2*	0.569	0.000507	0.0192
Pvr	-0.477	0.00103	0.0315	*Tbx21*	0.947	0.00121	0.0298
Rpl23	-0.651	0.00258	0.0435	*Tgfb2*	0.718	0.0027	0.0456
S100a8	1.64	0.00124	0.0315	*Tgfbr1*	0.466	0.000464	0.0188
Slc2a1	-1.15	9.51e-05	0.0096	*Tnfrsf11b*	-1.92	0.00101	0.0283
Spry4	-0.841	0.00247	0.0427	*Vegfa*	-0.97	0.00176	0.0358
Tbx21	1.06	0.000601	0.0315				
Tgfbr1	0.408	0.00219	0.0427				
Ticam1	-0.462	0.000903	0.0315				
Vegfa	-1.16	0.000457	0.0307				

Supplementary Table S4. Gene expression in VPA (left) and combination (right) treated mice compared to untreated mice.

**VPA vs control**				Combination vs control			
	**Log2 fold change**	**P-value**	**BH.p.value**		Log2 fold change	P-value	BH.p.value
Aldoc	-0.181	0.34	0.727	*Aldoc*	-1.06	1.05e-05	0.00244
Angpt2	-0.877	0.00323	0.438	*Angpt2*	-1.02	0.000925	0.0283
Axl	0.0954	0.631	0.883	*Axl*	0.676	0.00228	0.0418
Bnip3l	-0.218	0.136	0.632	*Bnip3l*	-0.773	1.61e-05	0.00244
Ccnd2	-0.176	0.491	0.817	*Ccnd2*	-0.897	0.00169	0.0358
Cd300a	0.225	0.403	0.776	*Cd300a*	0.977	0.00116	0.0298
Clec7a	-0.206	0.482	0.808	*Clec7a*	1.24	0.000278	0.0121
Ctsw	0.059	0.833	0.936	*Ctsw*	1.31	5.51e-05	0.00436
Ero1l	-0.855	0.009	0.438	*Ero1l*	-1.47	6.47e-05	0.00436
Hk1	-0.342	0.0149	0.438	*Hk1*	-0.438	0.00271	0.0456
Il18bp	0.294	0.116	0.632	*Il18bp*	0.88	5.27e-05	0.00436
Il22ra1	-0.0413	0.785	0.929	*Il22ra1*	-0.538	0.00179	0.0358
Irf3	0.147	0.126	0.632	*Irf3*	0.371	0.000593	0.0211
Itgal	-0.00782	0.977	0.992	*Itgal*	1.04	0.000736	0.0248
Itpk1	-0.546	0.00219	0.438	*Itpk1*	-1.25	7.35e-08	4.46e-05
Lilra5	0.0196	0.963	0.992	*Lilra5*	2.02	4.73e-05	0.00436
Mxi1	-0.27	0.173	0.645	*Mxi1*	-0.658	0.00241	0.043
Ndufa4l2	-1.07	0.00522	0.438	*Ndufa4l2*	-1.28	0.00123	0.0298
Nfkb2	0.09	0.38	0.765	*Nfkb2*	0.345	0.00218	0.0413
P4ha1	-0.271	0.121	0.632	*P4ha1*	-0.64	0.00097	0.0283
P4ha2	-0.581	0.00721	0.438	*P4ha2*	-0.915	0.00012	0.00664
Pck2	0.0551	0.557	0.867	*Pck2*	-0.339	0.0014	0.0315
Pdk1	-0.329	0.0657	0.55	*Pdk1*	-0.95	1.3e-05	0.00244
Prkca	-0.14	0.35	0.739	*Prkca*	-0.68	0.000134	0.00676
Rad51c	-0.145	0.353	0.742	*Rad51c*	-0.561	0.00131	0.0305
Rbl2	-0.0283	0.769	0.924	*Rbl2*	-0.339	0.00183	0.0358
Rpl23	-0.115	0.539	0.853	*Rpl23*	-0.908	6.44e-05	0.00436
Rps6kb1	-0.133	0.18	0.645	*Rps6kb1*	-0.367	0.00103	0.0283
Sfrp1	0.73	0.0873	0.618	*Sfrp1*	1.78	0.000251	0.0117
Srebf1	-0.172	0.105	0.632	*Srebf1*	-0.483	0.000103	0.00622
Stat2	0.428	0.00579	0.438	*Stat2*	0.569	0.000507	0.0192
Tbx21	0.764	0.00667	0.438	*Tbx21*	0.947	0.00121	0.0298
Tgfb2	0.364	0.101	0.632	*Tgfb2*	0.718	0.0027	0.0456
Tgfbr1	0.172	0.145	0.632	*Tgfbr1*	0.466	0.000464	0.0188
Tnfrsf11b	-0.394	0.436	0.79	*Tnfrsf11b*	-1.92	0.00101	0.0283
Vegfa	-0.452	0.111	0.632	*Vegfa*	-0.97	0.00176	0.0358
